# Exploring communication supports for children with visual impairment and blindness: A case study

**DOI:** 10.4102/ajod.v14i0.1620

**Published:** 2025-05-14

**Authors:** Kristen Abrahams, Dellicia de Vos, Armand Bam, Harsha Kathard

**Affiliations:** 1 Department of Health and Rehabilitation Sciences, Faculty of Health Sciences, University of Cape Town, Cape Town, South Africa; 2 Division of Student Affairs, Disability Unit, Cape Peninsula University of Technology, Cape Town, South Africa; 3 Small Business Academy, Stellenbosch Business School, Stellenbosch University, Stellenbosch, South Africa; 4 Inclusive Practices Africa Research Unit, Faculty of Health Sciences, University of Cape Town, Cape Town, South Africa

**Keywords:** visual impairment, blindness, communication, community, communication supports, home-based programme, multimodal, speech-language pathology

## Abstract

**Background:**

Early communication supports are essential for development, learning and later employment. For children with visual impairments and blindness (VI and B), we argue that communication and its supports need to be considered outside of the normative ableist framework to best facilitate development.

**Objectives:**

This study aimed to explore and describe how a home-based programme at a community-based organisation supported the communication development of children with VI and B by exploring and describing: (1) the organisation, its context and ethos; (2) the programme methodology including, role players, skills and activities; and (3) communication opportunities.

**Method:**

A case study design was employed, and data were collected through interviews, document reviews and observations. Notably, one member of the research team has a VI, which provided additional context and understanding of the case and enhanced the analysis process.

**Results:**

Key themes emerging from the data included the organisation’s history and context that shaped its ethos, the focus on a parent-led methodology and the support of communication through early multimodal opportunities.

**Conclusion:**

The findings emphasise the importance of understanding communication and communication supports beyond the normative ableist framework, which creates opportunities to appreciate and support communication holistically. More specifically, for speech-language pathologists, this study can expand their understanding of communication and raises questions about the profession’s potential contribution.

**Contribution:**

The study contributes to the literature within the South African context that demonstrates the value of communication and further captures how multimodal community support contributes to the health and wellbeing of people with disabilities.

## Introduction

Human communication is fundamental for socialising, learning and working (Littlejohn, Foss & Oetzel 2021). Through communication, we can create connections, develop relationships, engage with knowledge and earn an income. Early communication development provides the foundation for individuals to engage meaningfully with the world around them (Beuker et al. 2013). Children begin to learn important communication skills by engaging with their environment and interactions with people (Hoff 2006). The development of these early communication skills builds the foundation for later complex language and literacy emphasising the importance of early support for communication development.

For children with disabilities, the development of communication follows a unique trajectory compared to other children and subsequently, the communication supports required may vary in nature and complexity and may depend on the severity of the disability (Brouwer, Gordon-Pershey & Stransky 2023; Mosca, Kritzinger & Van der Linde 2015). When considering children with visual impairments and blindness (VI and B), communication development occurs with limited or no visual input (Mosca et al. 2015). Studies have argued that without appropriate stimulation, children with VI and B are at risk of communication delays (i.e. delays in speech, language and literacy development [Veldhorst et al. 2023; Vervloed et al. 2019]) because of their restricted interaction with their environment (Mosca et al. 2015) and the importance of vision for early communication acquisition (Rattray & Zeedyk 2005). Authors have therefore argued for the importance of early intervention to support children with VI and B to develop the necessary communication skills (Mosca et al. 2015). Subsequently, research has shown that programmes that promote early development and maximise the use of functional vision can make a difference in the long term (Dale, Tadic & Sonksen 2014).

A study by Lynch et al. (2018) evaluating the feasibility of a home-based developmental stimulation training programme supporting parents and caregivers of young children with visual impairment in Malawi found that providing families with support around VI and B positively impacted on both the developmental and educational opportunities. The findings of this study also emphasised the importance of collaboration across sectors as integral to the success of any early childhood initiative. Similarly, Dale et al. (2019) conducted a UK-based longitudinal observational study considering the effects of a home-based early intervention programme for babies and young children with VI. They found that children using the developmental journal with a structured development approach showed better outcomes than other forms of home-based support. Peltokorpi et al. (2023) argued early interactions between a child with VI and their parents may be affected by the parent’s lack of awareness of the importance of tactile experience for communication leading to fewer opportunities to develop their communication. They postulated that bodily-tactile modality (including body posture, movements and touch patterns) could be used to convey communicative intent. The study therefore aimed to test an intervention for children with VI and additional disabilities in parent-child dyads that can support increased access to social communication interactions through bodily-tactile expressions. The therapist facilitated mother and child intervention focussed on sharing ideas on how to support the child’s communication development. The results highlighted the importance of using bodily-tactile modality to support communication development in this population. Rattray and Zeedyk (2005) argued that the absence of vision alone does not prevent the establishment of a rich communicative system and has been conceptualised using theories that place an emphasis on the visual modes of communication and their importance for communicative interactions. Similarly, Peltzer-Karpf (2012) found that language acquisition for both sighted and participants with VI followed the same pattern of development, with a slower progression for children with VI which decreased with age and maturity.

Based on the literature, several observations were made: (1) the studies placed emphasis on the importance of early intervention to support children with VI and B and their families and highlighted the impact of late identification and support across the lifespan in terms of opportunities and growth; (2) while early supports are necessary for all children developing communication, we argue that the focus on the individual child for intervention is problematic in that there is limited consideration of the context of the child and their family and the influence of the environment on learning; (3) as communication is integrated with all aspects of a child’s development, its inclusion in developmental stimulation support programmes is critical highlighting the importance of using communication as means and ends; (4) interestingly, while the research has emphasised the importance of early support for children with VI and B, there is little emphasis placed on understanding the role of the speech-language pathology in supporting communication development in this population (Blackstone et al. 2021); and (5) we consider the contribution of Rattray and Zeedyk (2005) as seminal in signalling the need for change in our perception of communication and communication development. Rattray and Zeedyk (2005) argued that many studies have been conceptualised using theories that place an emphasis on the visual modes of communication and its importance for communicative interactions.

### Challenging ableism in communication

Drawing on the work of Henner and Robinson (2023), we draw inspiration from crip linguistics to unmask the pervasive and dominant understanding of communication in linguistics and other disciplinary fields. They argued that the ways in which languaging has been described contribute to the disordering of forms of communication falling outside of the dominant framing. Language, therefore, has been reduced to mainly speech and written systems which often excludes or devalues other forms of meaning-making such as gesture, art and touch. Disciplines therefore use disability as a way to categorise bodies as disabled by the way they produce language. This theoretical shift in perspective is an essential conceptual framing in approaching understanding VI and communication. Using this framing, we argue that the theory and normative basis for understanding communication uses an ableist lens that assumes that children are sighted and that communication partners are sighted. In this study, our assumption is that children with VI and B are capable of developing communication through an enabling environment. Using this framing, we have conceptualised communication as a multimodal, multisensory interaction in which people make meaning together. With this in mind, it would therefore be important to think about the different forms of communication for this study. Using an ableist framing, children with VI and B would not have access to visual forms of communication such as pictures, gestures and non-linguistic forms of communication which primarily convey extra-linguistic meaning. This narrow perspective may fail to acknowledge the *other* ways which support meaning-making that transcend visual mediums.

Extending our argument, not only are our understandings of communication (and what is valued) ableist but so are our everyday environments where children interact to make meaning. As such, children with VI and B are required to assimilate towards the normative standards of communication which requires them to live between sighted and blind worlds. In relation to this, Jenks (2005) reflected on parents experiences of raising children with VI and the negotiations of living in a sighted world. While there have been shifts in perspective around VI being a socially constructed disorder, Jenks (2005) argued that it does not negate the fact that the child with a VI still needs to negotiate a world which considers sight as a core value. It is within this context that we explore a community-based organisation and its contributions to supporting children with VI and B and their families.

### The contribution of speech-language pathologists to supporting communication for children with visual impairments and blindness

Speech-language pathologists (SLPs), with their focus on supporting communication, play a crucial role in supporting speech, language and literacy development. The profession has focussed on supporting communication disorders largely using a medical model which foregrounds assessment and management of discrete communication skills to improve quality of life and participation. While speech-language pathologists work with many different disabilities, little research has considered the contribution of supporting children with VI and B to develop communication (Carvalho, Fernandez & Montilha 2020). Additionally, speech-language pathologists are not specifically trained to work with this population (Mosca et al. 2015). While research interest in the field is growing, particularly around augmentative and alternative communication strategies for children with VI and B (Blackstone et al. 2021; Kavelin, Power & French 2024; McCarty & Light 2023), the depth and breadth of the research remain limited.

### Researchers

The research team consisted of two speech-language pathologists who were involved in the conceptualising of the study and integration of disciplinary knowledge and understanding of communication into the study. The third member of the team is an individual with a visual impairment who holds a Master’s degree in Disability Studies, and who managed the data collection process. The fourth team member was a representative of the organisation that was able to provide an understanding of the history, trajectory and context of the organisation itself.

## Methods

### Study context

Within a South African context, there is little understanding of the communication support for people with VI and B and therefore the study focussed on understanding the support offered to parents through an non-governmental organisation (NGO) called League of Friends of the Blind (LOFOB). League of Friends of the Blind, a community-based non-profit organisation (NPO), situated in the Western Cape, South Africa, provides support services to VI and B individuals and their families (LOFOB 2024). The organisation is the only one in the Western Cape with home and centre-based services for families of young children with VI and B. At the time of this study, LOFOB employed 23 people on a full-time basis. More specifically, the home-based programme involved a manager who was a professional occupational therapist, an additional occupational therapist, a social worker, a pre-school teacher, a teacher’s assistant and a driver. This unique combination of professionals and services made the site suitable for the exploration of communication supports.

### Aim and objectives

This study aimed to explore and describe how a home-based programme supported the communication development of children with VI and B. The objectives of the study were to explore and describe: (1) the knowledge, underpinning values and principles that inform the home-based programme; (2) the skills of stakeholders and role players in the home-based programme required to implement the home-based programme; (3) activities included in the home-based programme which supports communication; and (4) the opportunities available for the development of communication. Additionally, based on the insights gained through the case, the work further aimed to consider the contribution of SLPs to the VI and B population.

### Research design

The study utilised a case study design and followed the theory of Stake (1995) as this method uses qualitative data to answer pre-determined questions (Yazan 2015). By using a case study design, insight is gained into the particular situation of the participants, their circumstances, social relationships and the practices that are embedded in them (Taylor, Bogdan & Devault 2015). While LOFOB has many offerings, the case specifically focussed on the home-based programme from its inception which acted as the bounding for the case. Through the study, it was important to document what has been performed in communities to support communication for children with VI and B in order to strengthen our efforts towards inclusion. Data collection for the study took place during the coronavirus disease 2019 (COVID-19) pandemic which had significant impacts not only on the data collection process but also the contributions of the home-based programme. These nuances will be highlighted throughout the article.

### Participant recruitment

Participants in the study were categorised into two groups. Group one was made up of three LOFOB staff members namely the programme manager (Lydia), the social worker (Isaac) and the occupational therapist (Sally) who were currently working at the organisation and were involved in some capacity in the running of the programme. The second group was made up of four parents whose children participated in the home-based programme. Two of the parents were mothers (Gertrude and Mary) and the third and fourth were a married couple (Hilton, Mavis and daughter Samantha). Pseudonyms are used throughout. At the time of the study, the children of the parents interviewed had moved to different primary schools. See Table 1 for further details about the participants. Purposive, non-random sampling was implemented in this study. This method allows for the collection of data that is specific to the study aims and allows for participants with an in-depth understanding of the phenomenon to be recruited (Emmel 2013).

**TABLE 1:** Participant descriptions.

Pseudonym	Role	Description
Lydia	Programme manager	Occupational therapist by profession has worked in organisations in different capacities for 25 years
Isaac	Social worker	Working for the institution for 3 years, involved with psychosocial support for children and parents
Sally	Occupational therapist	Had worked for the organisation for 1 year in the home-based programme
Hilton, Mavis	Parents of Samantha (child with VI)	Daughter (13 years old) took part in organisation programmes
Gertrude	Parent of a child with a VI	Child (6 years old) took part in organisation programmes
Mary	Parent of a child with a VI	Daughter (12 years old) took part in organisation programmes

VI, visual impairment.

### Procedure

Following ethical approval, LOFOB was contacted by the researchers for organisational consent. The recruitment of participants was facilitated through engagements with the programme manager to identify interest in the study among current programme staff members and caregivers. Following this, the researchers made initial contact with the participants via email or via a telephone call. Once identified, participants were given information sheets and consent forms for written and/or verbal consent before data collection because of the COVID-19 restrictions. Data were collected using a number of different methods including organisational documents, observations, field notes, the researcher’s reflective diary and semi-structured interviews allowing for triangulation of findings (Ahmed 2024). The researchers also drew on insights and understanding from another study conducted at the same organisation which focussed on a different offering namely the centre-based programme (Kamedien 2023).

### Ethical considerations

Ethical clearance was obtained from the Faculty of Health Sciences’ Human Research Ethics Committee at the University of Cape Town on 07 October 2021 (No. 586/2021). This study adhered to the Declaration of Helsinki (World Medical Association 2013) and upheld the ethical considerations of beneficence, non-maleficence, justice and autonomy as described throughout the methodology. For autonomy, participants were provided with an information letter and consent form to review before confirming participation. Participation was voluntary and the participants could withdraw at any point, without penalty. For confidentiality, contact details were deleted following the completion of the study and pseudonyms were used throughout. While there was no direct benefit for participants, they were informed that the study could help strengthen the organisation’s offering. Precautions were taken to ensure non-maleficence with relevant referrals to support as necessary.

### Data analysis

The data analysis process was guided by Braun and Clarke’s (2006) six-step framework for thematic analysis to assist with identifying common ideas and patterns of meaning for analysis and representing the findings. The analysis was driven by the data that were captured so themes were not pre-determined. The following steps were used during the analysis process: (1) the researchers individually read and re-read the transcripts to familiarise themselves with the data; (2) each researcher made notes about key ideas of the data that stood out to them in line with the aim and objectives of the study; (3) collectively the researchers discussed these key ideas to identify emerging themes in the data; (4) the researchers consolidated the themes by extracting the relevant data that supported the themes; and (5) as a collective, the themes were reviewed and refined. The research process was further enhanced by the contributions of a team member with a visual impairment, who conducted the interviews and assisted in the analysis and identification of themes. This contribution strengthened the data collection and analysis process as it allowed for deeper understanding and sense-making of the data through her own lived experiences. In particular, we drew on reflexivity within this process to reflect on our personal understanding and orientation to communication as a basis for the analysis and interpretation (Olmos-Vega et al. 2023).

### Scientific rigour

Scientific rigour was ensured during the study through considerations of transferability, creditability, dependability and confirmability. The researchers aimed to uphold trustworthiness by presenting data transparently, truthfully and accurately (Kuper, Reeves & Levinson 2008). Transferability was achieved by providing descriptions of each participant, the setting and the research methodology used. Confirmability was achieved by keeping an audit trail of all participants’ recorded interviews and verbatim interview transcripts. Credibility and dependability were ensured through member checking and the contributions/insights of a research assistant with VI.

## Results

### Introduction to the case

The case study focussed on an organisation called LOFOB which is a non-governmental organisation which focusses on supporting individuals with visual impairments and blindness. From its inception, the founding member of the organisation was a blind man who played a crucial role in the leadership and management of the organisation. Since then, the organisation has transitioned from a focus on charitable giving towards independence development which has grown to include orientation and mobility, social work, occupational therapy, sports, hostel accommodation for both males and females, social enrichment programmes and a professional administrative team. In the 1980s, LOFOB introduced its early childhood development programme the first of its kind in the Western Cape. Today, LOFOB largely serves marginalised communities as part of its offering. The findings specifically focus on the home-based programme where we explore the key themes emerging including its ethos, home-based methodology and supporting communication.

### Ethos of the programme

The ethos promoted by LOFOB was evident in the interviews with parents, staff and leadership and the document analysis. The ethos of the organisation challenged normativity; shifted focus to capabilities, independence and functionalities; and adopted a lifespan focus.

#### Challenging normativity: ‘Strength not to feel OTHERwise about my child’

The parents said:

Mavis: ‘You know I hated the word “disabled” or “normal”.’

Hilton: ‘We don’t use normal.’

Mavis: ‘Yeah which was quite a learning.’

Gertrude: ‘Having a blind child, it was a death sentence or end of the world … but you learn to see a life with her … you can still treat her like others. So that is where I gained the strength that let me not feel otherwise about my child. Let me take my child as a special person and I must take her as a gift because God gave me that gift. So that was the strength that I got from LOFOB.’

Through the words of the parents, it is clear that challenging the idea of normativity was a key learning and insight that they gained through their engagements with LOFOB, indicating the emphasis on understanding the unique capabilities of their child beyond their visual impairment.

#### Education is key: Responding to stigma and discrimination, exclusion

The ethos of ongoing education is embedded in the organisation. League of Friends of the Blind invested in the education of parents which carries through to their children and to community members they are in contact with:

Hilton: ‘We actually make her strong enough to cope with all of the stigmas and the cruelty. You know when we used to go to a restaurant the kids would gather around her in the play area and they would like want to ask her questions and or they’ll say she scares them. We didn’t stop her from playing with other children because we knew there was always going to be another scene that she was going to be upset with. Then the one day she comes to us she says you know daddy that one child asked me ‘are you blind’ so I told her no, you can see I’m not blind, I’m partially sighted and that to her was … so what she did good After a few visits we liked going to the restaurant.’

Hilton: ‘She [*Mavis*] just goes automatically into education mode. My mum does the same when people stare at Samantha.’

Sally: ‘Bring them out of their homes because we’ve noticed that a lot of community members aren’t aware that there’s someone with a visual impairment living in their area.’

The LOFOB environment supported parents in challenging the deficit norm for children who were blind and VI. The realisation that the imposed social norm was constructed in an ableist society helped to disrupt the notion of being normal and therefore develop an attitude towards supporting their children to learn in ways which were helpful for them. Both the organisation and parents used ‘education is key’ in everyday situations to disrupt stereotypes and to educate – an ethos in which families and children are also actively involved. Challenging normativity through education sets the stage for shifting the focus to developing capabilities which was actively promoted in the programme.

#### Shifting focus to capabilities, in(ter)dependence and functionality

The parents and LOFOB team concurred that the programme shifted the focus to capabilities, in(ter)dependence and functionality which helped to capitalise on children’s and parents’ strengths and facilitate positive attitudes and tangible everyday outcomes:

Hilton: ‘LOFOB just made us realize actually there’s just nothing wrong with her. She’s just differently abled.’

While it was a process, like it is for all children, parents were motivated by their children’s progress which was often beyond their expectations:

Hilton: ‘When the doctor gave us the diagnosis he said ‘the middle brain is missing’ It means that she’s possibly gonna have mental impairments and there’s gonna be coordination problems because her left and right side will never align … I promise you, we’ve never seen that at all. So as much as that was her diagnosis and that’s what the scans showed and the MRI showed (but) we’ve never been able to say that Samantha is lacking from a mental capacity ever.’

Hilton: ‘One week the kids were the servers so that built their confidence but I think the reason that they brought us (parents) in to come and observe Samantha was that just to dispel misconception that she’s unhappy here, she’s quiet and when we saw Samantha it was a different kid. She was serving lunches, she was assertive, she was taking the lead on things.’

Mavis: ‘I remember LOFOB used to call us out on that and say, you need to let her sort herself out, she’s got to be independent. Don’t dress her, let her do as much as she can possibly because you need to enable her and by you taking over all of that, you’re actually disabling her. They used to scold us a lot.’

#### A lifespan focus

The data confirmed that LOFOB has a lifespan approach with early intervention being a key focus. They support critical transitions, for example children’s entry from hospital to homes, from home to preschool, to primary and high school and post-school supports. In relation to the home-based programme, there was a specific emphasis on supporting early transitions for children with VI and B and their families which evolved over time:

Isaac: ‘The sooner that we are able to work with a baby and a mom of a baby, the better. My aim is I would say to prepare the child for life.’

Issac: ‘So its a start for them to be … moving up to be able to be in LOFOB ECD [*early childhood development*] centre. … so you get supported while you still in … [*I*]t’s an early intervention for LOFOB for parents … Then they are prepared for ECD and primary school up to high school. At LOFOB, it’s a foundation, I’d say it’s laying a foundation for them.’

Sally: ‘Providing the stimulation and the three-monthly check-ins with the parents uhm to get updates on their development. What’s new, what’s happening in their lives, what development has happened within the last three months. What stimulation they can provide within the home and then if they are attending a special care centre, also providing support to those teachers.’

### A parent-led methodology

The LOFOB home-based programme is underpinned by a parent-based methodology which includes several support structures including home and centre visits, individual and group support, professional support, psychosocial, parent support and resources. The LOFOB home-based programme is aimed at guiding and supporting parents with newly diagnosed blind and visually impaired babies and children to cope with raising a child with special needs. This programme is facilitated by professionally trained LOFOB staff members and may take place at the homes of clients, at the LOFOB centre or at community centres. Each client (baby or child and parents or caregiver[*s*]) is assessed individually before joining the group sessions. During the group sessions parents support and encourage each other through sharing their experiences and the LOFOB staff support them through encouragement and by providing access to necessary resources. For the parent-led methodology, we focus on the key methodologies informing the programme including home-based support, resources including staff and services offered.

#### Contextual shifts necessitating changing focus

The focus of the home-based programme was to provide parents with the necessary support within the different spaces that they occupy within their communities:

Lydia: ‘What is important for us as providing the support service to children uhm that is their space, that is their home so we were very much about where you are as the family … to use your own environment, to look at your own support system because that will then make sense to work from there from the inside-out …’

Lydia: ‘… when I say home support it can be at home, it can be … the young blind child is attending a crèche or whatever else, we will go there you know.’

While conceptualised as home-based, the programme has evolved into a centre-based programme because of contextual changes such as the COVID-19 pandemic and increased safety concerns with going into communities:

Sally: ‘… our home program, … initially started out pre-COVID, as visits where we would go to the clients’ home, do assessments give them home programs, stimulation programs where we would see what the client presents with and then giving parents the necessary tools to implement stimulation within their home context

Lydia: ‘… COVID has impacted a big deal … We were so so far away from our parents and our families. Children were still getting referred to our organisation … we had some contact but we didn’t see physically … we brought in videos some of the moms were able to send through videos …’

Sally: ‘Uhm since COVID and with the safety being an issue … one of our drivers were nearly hijacked. Uhm we’ve now changed it a little bit to having the parents either access and come to LOFOB like an out-patient … kind of a program uhm where we see them here otherwise if they are very far and they not by means to come here, we do meet them at a clinic or uhm like … a district hospital or some sort of community centre uhm within their community.’

#### Supports offered in programme

Through its programme, LOFOB offers a number of supports including individual support for families, psychosocial support through the social worker and parent or caregiver support through the parental support group.

**Individual support:** Initially, the LOFOB team would focus on providing individual support within the home environment:

Sally: ‘We would go to the clients’ home, do assessments uhm give them home programs, stimulation programs where we would see what the client presents with and then giving them the necessary tools and the parents the tools to implement stimulation within their home context.’

**Psychosocial support:** All the parents were very appreciative of the support they received from the LOFOB staff and from the parent support group:

Gertrude: Having a blind child it was a death sentence or end of the world. You can see it … [*Y*]ou can see a life with her … [*Y*]ou can still treat her like other people, and you must know that if you have a blind child, it’s a blessing from God. That’s not a sin or a curse so that’s the strength that I got from them because as from my side I was asking too many questions to God. How can He give me the child like this, how am I going to raise this child or to manage to look after this child?

Lydia: ‘… I always say, in order for the child to be ok, the parents needs to be ok.’

Isaac: ‘… I do psycho-social support for the parents as they need it mostly at that stage because most of them … are still … going through a lot of things, pressures from the family, in denial about their child’s visual impairment and so there’s lot going on there. Psycho-social support linking them to services like resources that they might need.’

**Parent and caregiver support:** The parents felt that they received support from one another during the group sessions and from LOFOB staff:

Hilton: ‘… we [*parents*] were actually good support to one another in that regard. … we could share a lot and help one another with things …’

Gertrude: ‘… like they [*LOFOB staff*] teach us as a parent … how to communicate with your child … the way you must treat your blind child, you mustn’t treat her specially like she’s special like she’s a special needs yes she is a special needs but don’t give her that attention. She must be like the other children around her.’

Lydia: ‘… parents can get together in a room like this and just feel that this is a safe space to say oh so this happens to you as well. How did you ma we talk about it, we laugh about it, we cry about it … parents support group facilitated by both occupational therapist and social worker.’

Lydia: ‘… to make sure that the child is developing optimally and then also to make sure that the parents are equipped with the information and the knowledge and the resources that they need to make sure that the child is as ok as what the child needs to be … looking at ages and stages and stuff and so just to say that LOFOB provide support to children that have [*multiple*] impairments.’

Sally: ‘I work predominantly with the home-based program. Uhm so doing developmental stimulation and programs with the parents who come in for out-client care …’

Gertude: ‘… but [*with LOFOB*] I was having a parent group I’ve learn a lot. So that is where I gained the strength that let me not feel otherwise about my child.’

### Supporting communication

League of Friends of the Blind’s ethos and methodology of practice shaped the opportunities for communication support in the programme. Communication was supported in the following ways: (1) creating *opportunities* for communication both directly and indirectly; (2) *multimodal* communication and (3) an emphasis on *early* exposure.

#### Creating opportunities for meaningful engagement to support communication development

The importance of creating opportunities which supported early language and literacy development in children was highlighted, with particular emphasis being placed on appreciating how children with VI and B may experience the world differently:

Lydia: ‘Children who are sighted are exposed to literacy and to early literacy in everything you know because the world is not geared to include children who are visually impaired. I mean there are just right now in this room, there’s so much printed information. There’s pictures, there’s formats, there’s photographs … I always use the example of the little the child that even before the age of two or three already rides past the big M for McDonald’s knows already that’s where I can get my ice cream or whatever else because I see that big M and you know the child with a visual impairment, they miss out on that.’

Using this understanding, emphasis is therefore placed on creating a supportive environment that can foster holistic development through training and supporting caregivers to create opportunities for engagement. The importance of every day doing in shaping communication is emphasised. These foundations of early support in the environment create opportunities for children to succeed later in life:

Hilton: ‘The one thing that they told us not to do, was to leave Samantha in a corner type of thing, to her own devices. [*Mavis*] was mentioning earlier [*in the interview*], if she starts rocking, it’s a sign of boredom … we should actually try and work with her instead of just leaving her to do her own thing.’

While there was a direct focus on communication in some cases such as the early exposure to Braille, the main way in which communication was supported was through engaging in occupations such as play that supported and reinforced communication through doing:

Lydia: ‘The importance of a program such as LOFOB [is] to kind of empower parents with the information [*like*] “your child needs to play mommy”. “But I don’t know how to do that with my child” so therefore we help and we give you support programs and information in terms of, this is what play with your child needs to look like.’

Sally: ‘Whether it’s you getting pictures or uhm if they have some kind of remaining vision or whether it is just speaking to a child a lot. Uhm explaining what’s happening, what’s around them, what’s going on, who’s in the room, what things look like, what things feel like. Having the child explore their environment as much as possible but I think that’s kind of what I try and do as much as I can.’

#### ‘Communication is more than words’

For children with VI and B, the importance of multimodal, multisensory communication support through play was emphasised. A combination of oral, tactile and visual (using residual vision) input was emphasised. Using thick description during communication interactions through engaging the senses of hearing (i.e. providing an in-depth description of an activity), in combination with touching and feeling placed emphasis on providing the child with rich communication input. In addition, multiple modalities were used including reading, technology, pictures, videos, objects and songs:

Isaac: ‘With visual impairment, more talking, more explanation, more description of things because most of them can barely see.’

Lydia: ‘We often say to parents hold your child close. I’ve had parents that take the child’s hand when you talking especially when your child is totally blind or severely visually impaired so that he’s able to feel where the sound comes from and then take his hand back to his mouth and bring it back to your mouth because he’s then able to hear and to feel [*the sound*].’

Mary: ‘The ball with the thing inside where you can throw it and then she can hear which side did it go to and then she would follow that ball and pick it up.’

Gertrude: ‘Mostly to watch TV and also to listen to the radio.’

Mavis: ‘And then eventually you know you get a child a tablet or something like that you know for her to learn and she was on YouTube which I think also helped because there was a lot of educational stuff in the cartoons itself that she picked up and that I realized this is actually helping us so much.’

Mavis: ‘We used to play songs for her.’

Hilton: ‘And then she’d dance yeah.’

Mavis: ‘As soon as she could walk and then she would dance and if she wanted her daddy to play another song then she’d say another number, another number and then she’d dance.’

In particular, tactile exploration of the child’s environment is encouraged as an important precursor for literacy development for learning Braille:

Lydia: ‘We’ve got adaptive books that we lend out to our families and it can be the tactile books in terms of introducing them to so we sit and then we take our parents through that.’

Mary: ‘They would sit and tear up papers and play with beads and different types of textures and stuff. That was also like, you don’t realize that your child needs these things especially from a visually impaired type of child so that also they said will help eventually with them reading Braille.’

Gertrude: ‘The eggshells. We used to play with eggshells so that she can know the Braille. It’s six eggs neh and then that’s how they learn the Braille. We make the dots by [using] the eggshells and then you put in the shells there inside then that’s how they learn while they are playing.’

Gertrude: ‘I was doing the reading before she goes to sleep. I was doing the story books and also she was registered in the library for the blind. So they use to send the books and something called the Braille reader where you put in the CD and then it will read for her with different languages. That’s how she learnt.’

#### Early support leads to later success

Creating opportunities for early development of necessary skills for learning was emphasised by participants, with particular emphasis on how such early supports can have an impact on their activity and participation as they get older:

Isaac: ‘If you learn to communicate at a young age it will have more positive results for the child, their self-esteem, their image and how they grow up, their confidence and how they do things in later in life.’

Lydia: ‘We are very proud that we introduce our little ones as young as two-and-a-half years of age to Braille.’

## Discussion

The discussion will focus on the main themes emerging from the study integrating the potential implications for speech-language pathology practice.

### Early support as a foundation for the future

The importance of early support as a basis to build a strong foundation for later learning was a key principle informing the development of the programme and the overall ethos of the organisation. This was evident in the ways in which participants reflected on the programme:

Isaac: ‘At LOFOB, it’s a foundation, I’d say it’s laying a foundation for them.’

This early support is realised through focussing on parental engagement and support so that parents and caregivers are able to support their child’s development in the home before entering the formal schooling system. Through this mechanism, LOFOB supports children and their families through a critical period of rapid child development and functions to support early transitions from the home to more formal schooling through their home-based programme.

The principle of early support is coupled with a focus on capabilities, independence and functionality which is clearly emphasised through the perspectives of the leadership, staff and parents. The movement away from the focus on disability towards the capabilities signals an intentional emphasis on using the strengths and capabilities of the child to support overall development (Mitra 2006). For communication, it therefore signals a movement away from focussing on vision towards other modalities that are central to supporting the development of communication for children with VI and B (Rattray & Zeedyk 2005). This perspective is consistent with the movement in disability studies that challenge the disothering of otherwise forms of doing and being (Henner & Robinson 2023).

White Paper 6 which focusses on building an inclusive education system acknowledges that all children learn differently and places emphasis on providing support at various levels, using different methods, to maximise the participation of all learners (Department of Education 2001). In working towards inclusive education, what should integration look like? Do learners require intensive teaching to lay a foundation for later learning – that is building early capabilities to negotiate a sighted world as a prerequisite for an inclusive education model? Through supporting these critical transitions for children, LOFOB is seeking to bridge the gap between the child’s capabilities and the expectations of the schooling system. An important part of realising the goals and values of White Paper 6 is the conscientisation of the dominant ableist framings embedded in the schooling system that make integration a challenge (Walton & McKenzie 2020). In extending this support, there may be further opportunities for LOFOB to support other critical transition periods for children like between primary school and high school.

### Supporting communication through occupation

Participants spoke about the importance of engaging children in exploring their environment through multimodal experiences, with particular emphasis on allowing the child to engage in multiple occupations within their daily lives. The participants reflected on the importance of play as a means to support growth and development. Communication, as an integral part of engaging in occupations like play, was therefore embedded as part of the programme through its focus on allowing the child to explore their environment. As humans, to engage meaningfully in occupations (ways of doing) in everyday life, communication is central (Peters et al. 2023). Communication was therefore more covertly supported through occupation, particularly with the early communication supports.

Interestingly, exploring communication through doing, in which the participants placed emphasis on engaging with the activity itself, drew into sharper focus the importance of alternative forms of communication that are deeply meaningful for children with VI and B. A significant emphasis was placed on understanding how exploring objects through a combination of tactile, olfactory, visual and auditory cues allowed the child to develop an understanding of their environment. While the literature focussing on visual impairment and blindness has placed emphasis on tactile modalities (Peltokorpi et al. 2023; Rattray & Zeedyk 2005), the study highlights the importance of using multiple means of sense-making in shaping and supporting communication. Such learning, we argue, is not only beneficial for our understanding of communication development for children with VI and B but can also enhance the kinds of support offered to all children. Shifting the dominant perspective on communication through a sighted world towards an emphasis on languaging and valuing all that helps us to make meaning (Henner & Robinson 2023) has the potential to strengthen communication for all.

Beyond the tactile, visual and verbal modalities highlighted in the findings, parents alluded to the importance of songs, radio and music for their children. While not a central feature of the parent narratives, we argue for the importance of music as a way to support early learning, interaction and connection (Metell 2015) which can provide opportunities for children to make sense of their environment. The importance of music for meaning-making can be strengthened through the LOFOB programme and for SLPs, it signals an opportunity to use music as a means to support early communication development (Knight & Rabon 2017).

More overt emphasis was placed on communication when preparing children for early literacy development where participants discussed the early tactile supports necessary to support later literacy development. We argue that more overt exposure and support for communication, particularly early supports, could be programmed into the practice to further build on the current communication supports offered through the programme. For example, SLPs could work with organisations to strengthen their organisational capacity for programmatic support for communication development through occupation as opposed to solely focussing on direct intervention.

### Strengths and limitations

Of particular strength for the study was the inclusion of a researcher with visual impairment in the data collection, analysis and write-up of the article. Using her positionality and reflecting on her experiences, she was able to provide deeper insight into the key aspects of communication and its support. We acknowledge the impacts of COVID-19 on the data collection process which limited access to participants and had significant impacts on the core contributions of LOFOB’s home-based programme. We are therefore acutely aware of the contextualised nature of the findings and emphasise the importance of understanding the findings in relation to the social impacts of the pandemic.

### Implications

For the implications, we reflect on the meaning and contribution of the article for SLP. For communication, a focus on capabilities illuminated the importance of multimodal, multisensory supports through occupation as central to development for children with VI and B. These findings are significant as they signal the importance of appreciating the complexity of communication highlighting that for all children supporting communication goes beyond visual and written modalities. For the profession of SLP, it signals an opportunity to expand our approach to supporting communication to include other forms, shapes and mediums of communication in our practices.

## Conclusion

The study documented a home-based programme for children with VI and B with specific emphasis on how communication was supported through its offerings. Through the case study, it was clear that the ethos underpinning LOFOB’s commitment and contribution to the vision-impaired community significantly influenced its key stakeholders, the activities and services offered and the opportunities for communication. The emphasis on capabilities, in(ter)dependence and functionality and challenging normativity, echoed through the contributions of parents and were evident in the structure and focus of the individual and group engagements.
